# Appreciating the role of cell shape changes in the mechanobiology of epithelial tissues

**DOI:** 10.1063/5.0074317

**Published:** 2022-03-16

**Authors:** Marine Luciano, Marie Versaevel, Eléonore Vercruysse, Anthony Procès, Yohalie Kalukula, Alexandre Remson, Amandine Deridoux, Sylvain Gabriele

**Affiliations:** University of Mons, Interfaces and Complex Fluids Laboratory, Mechanobiology and Biomaterials Group, Research Institute for Biosciences, CIRMAP, 20 Place du Parc, B-7000 Mons, Belgium

## Abstract

The wide range of epithelial cell shapes reveals the complexity and diversity of the intracellular mechanisms that serve to construct their morphology and regulate their functions. Using mechanosensitive steps, epithelial cells can sense a variety of different mechanochemical stimuli and adapt their behavior by reshaping their morphology. These changes of cell shape rely on a structural reorganization in space and time that generates modifications of the tensional state and activates biochemical cascades. Recent studies have started to unveil how the cell shape maintenance is involved in mechanical homeostatic tasks to sustain epithelial tissue folding, identity, and self-renewal. Here, we review relevant works that integrated mechanobiology to elucidate some of the core principles of how cell shape may be conveyed into spatial information to guide collective processes such as epithelial morphogenesis. Among many other parameters, we show that the regulation of the cell shape can be understood as the result of the interplay between two counteracting mechanisms: actomyosin contractility and intercellular adhesions, and that both do not act independently but are functionally integrated to operate on molecular, cellular, and tissue scales. We highlight the role of cadherin-based adhesions in force-sensing and mechanotransduction, and we report recent developments that exploit physics of liquid crystals to connect cell shape changes to orientational order in cell aggregates. Finally, we emphasize that the further intermingling of different disciplines to develop new mechanobiology assays will lead the way toward a unified picture of the contribution of cell shape to the pathophysiological behavior of epithelial tissues.

## I. INTRODUCTION

**FIG. 1.**
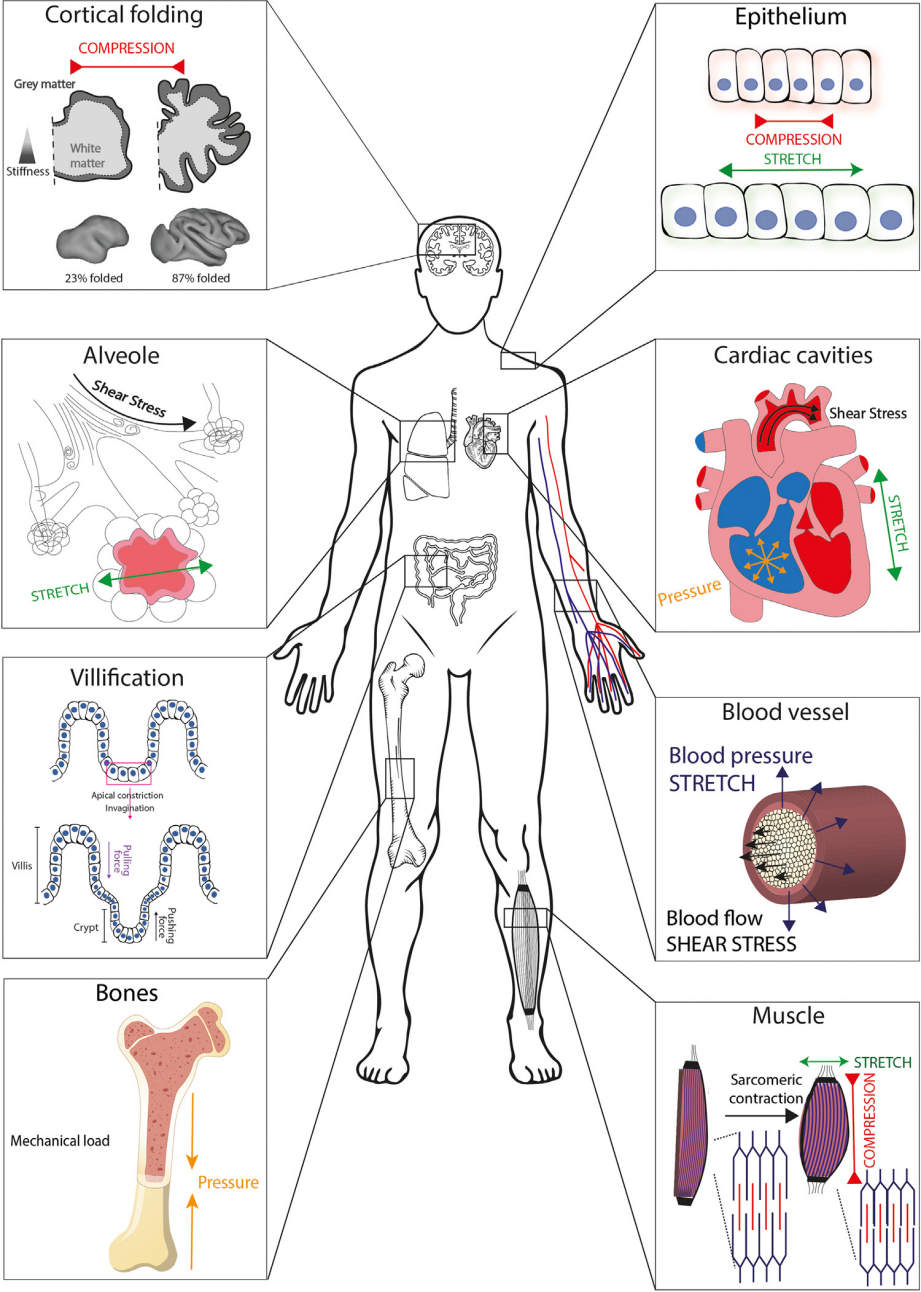
Illustration of tissues that experience mechanical strain and external forces in the human body. Human tissues are exposed to different types of forces such as tension, compression, fluid shear, pression, and stretch. Cells and tissues are, therefore, able to sense strain, stiffness, and other mechanical parameters associated with forces.

Forces are ubiquitous *in vivo*, and a large amount of evidence has accumulated to illustrate that epithelial cells live in a microenvironment where forces and mechanical constraints shape their morphology and influence their biological function (Fig. 1).^1–3^ Growth-induced confinement and compression were shown to lead to the buckling of epithelial tissues,^4^ which is implicated in the formation of folds, as observed in the *Drosophila* wing development.^5^ Although epithelial folding has mainly been attributed to mechanical forces generated by the apical actomyosin network, it was shown recently that a local decrease in basal tension and an increased lateral tension could drive complex three-dimensional morphological changes, such as the formation of folds in developing *drosophila* wing imaginal disks.^6^ The gyrification of the human brain is another beautiful example of three-dimensional patterns produced by mechanical forces.^7^ Indeed, gyrification, which enables an important increase in the surface of the cortex by the formation of folds in the thin layer of gray matter,^8^ was found to arise from a simple mechanical instability driven by the tangential expansion of the gray matter, which is constrained by the white matter.^9^ Although the tangential expansion of the cortex is determined by many molecular determinants, it was shown that the size, shape, placement, and orientation of the folds arise through iterations and variations of an elementary mechanical instability modulated by early fetal brain geometry.^10^ Other organs of the human body are also shaped by mechanical forces to maximize their functions, such as the small intestine, which has developed a folded structure called villi to increase the mucosal surface area.^11^ The formation of villi and crypts in the intestine is related to tissue invaginations that result from apical contractions of epithelial cells.^12^ These folding and buckling mechanisms lead to the formation of topographies, which are specific to each organ or tissue with ducts, hollows, and bumps.^13,14^ Curved features, which are found in blood vessels, lung alveoli, intestinal villi, or lumens, spread over a large scale, from a few nanometers up to hundreds of micrometers in radii and are directly related to the physiological function of the tissue.^15^ For example, the folding of the intestinal epithelium, in the form of villi, results in a controlled localization of biological signals and leads to the sequestration of intestinal stem cells at the base of the villi.^16^ In addition to these physiological processes, some pathological situations were also associated with forces and mechanical constraints that lead to significant cell shape changes. Major modifications of curvature and shape were observed, for instance, in keratoconus, which is a potentially sight threatening corneal disease characterized by a cone-shaped protrusion on the anterior corneal surface.^17^ Abnormal folding patterns were observed in a diverse array of neurodevelopmental disorders, underlining the clinical relevance of understanding the role of mechanical constraints and associated cell shape changes.^18^ Recently, imbalance between apical and basal mechanical tension and tissue curvature was suggested as key determinants of epithelial tumorigenesis in various organs.^19^

These examples suggest that cells and tissues must deal with forces and mechanical constraints to maintain their “mechanical homeostasis” (Fig. 1). The field of mechanobiology has emerged at the interface of biology, engineering, and physics based on the recognition that physical forces and changes in the mechanical properties of cells and tissues contribute to development, cell differentiation, physiology, and disease.^20^ In addition to the role of forces in shaping tissues and organs, mechanical stresses acting on cells and tissues also contribute to modifications of their functions. It is now well accepted that the blood flow applied by hemodynamic forces on the vessels wall plays a major role in shaping the vasculature.^21,22^ Shear force changes are sensed by mechanosensors, such as plexin, which are present at the surface of endothelial cells. In turn, these mechanosensors can activate signaling pathways to modulate an atheroprotective phenotype in regions of static laminar flow or a proatherogenic phenotype in response to disturbed flow.^23^ Endothelial cells respond to blood flow modifications by rapidly and reversibly polarizing, elongating, and aligning in the direction of flow, demonstrating a key role of their shape changes.^24^ As demonstrated in the zebrafish embryo, flow conditions are involved in cancer cell extravasation by playing both on the ability of cancer cells to arrest and adhere to the endothelium and on the remodeling of endothelial cells to help cancer cells exclusion.^25,26^ In lungs, air flow produces shear forces on the alveolar wall, and pulmonary cells are subjected to strain during inhalation, leading to an increase in ∼25% in the circular circumference of capillaries.^27^

It is now clear that mechanical constraints and the resulting changes of cell shape play a crucial role in maintenance of the cell homeostasis and emergence of specific diseases. Consequently, cellular mechanotransduction—the molecular mechanism by which cells convert mechanical forces into biochemical activities—is intensively studied, and significant advances have been made over the last two decades. However, major challenges remain regarding the understanding of the regulation of gene expression in living cells and tissues that experience physical forces. Here, we review relevant works that integrated mechanobiology to elucidate some of the core principles of how cellular geometry may be conveyed into spatial information to guide collective processes in epithelial tissues, and we describe recent insights into the role of cell shape changes in epithelial mechanotransduction processes. We will introduce the basic mechanisms linking the cell morphology to its cytoskeletal organization and how the modifications of cell morphology regulate mechanotransduction pathways. We will present accumulative evidence from mechanobiology that highlights the role of forces and tension for shaping tissues during morphogenesis, and we will discuss how actomyosin forces are exerted and distributed during morphogenesis to allow a dynamic remodeling of epithelial tissues. We will then show how intercellular adhesions play a crucial role in remodeling cell shapes and force transmission between cell assemblies due to their connection to the actin cytoskeleton. We will introduce recent efforts that exploit physics of liquid crystals to connect cell shape changes to orientational order in epithelial cell aggregates. Finally, we will present advanced bio-engineered platforms that are developed to explore and decipher key mechanobiological mechanisms involved in the physiopathology of epithelial tissues.

### A. Cytoskeletal regulation of epithelial cell shape

Living cells must change their shape dynamically during many important physiological processes, such as division,^28^ migration,^29^ and differentiation.^30^ In the early embryo, the cell cytoskeleton is dynamically remodeled with unique spatial and temporal precisions to drive developmental processes in response to changes of the *in vivo* microenvironment.^31^ One of the most studied and yet less understood processes in cell biology is the generation and maintenance of eukaryotic cell shape. The current view is based on the role of the cell cytoskeleton, which plays a specific organizer role in the cell shaping process. In addition, the cytoskeleton carries out important functions by physically connecting the cell to its microenvironment, by coordinating forces and enabling the cell to move and adapt its own shape.^32^

Eukaryotic cell shape changes are mainly determined by the spatial reorganization of each cytoskeletal component (actin microfilaments, microtubules, and intermediate filaments), which form altogether a dynamic and responsive network.^33,34^ Actin filaments are polarized polymer filaments that interact with myosin molecular motors to generate contractile forces.^35^ Microtubules are another type of polarized polymers, which interact with the molecular motors of the dynein or kinesin families. Myosin, dynein, and kinesin molecular motors have essential roles in organizing dynamically the cell cytoskeleton. The spatial reorganization of the cytoskeleton allows cells to resist to moderate mechanical constraints.^36–38^ Furthermore, cells can also use their cytoskeleton to exert traction, contraction, and protrusive forces.^39–42^ Cytoskeletal forces range between pN and nN and can be produced by polymerization or depolymerization of actin and microtubules.^43^ Indeed, it was shown that growing microtubules can exert forces between 0.5 and 5 pN,^44^ while stall forces of polymerized actin filament were estimated in the 1–10 pN range.^45^ Interestingly, actin filaments are much less rigid than microtubules. Their persistence length (lp), which is a measure of the filament flexibility that increases with stiffness, is ∼17 *μ*m, whereas lp ∼5 mm for microtubules and lp ∼0.3 *μ*m for intermediate filaments.^46^ Microtubules are, therefore, the stiffest filament of the cytoskeleton and can resist to large forces, although they can buckle under large compressive loads in cells.^47^ Intermediate filaments can be crosslinked to each other as well as to actin filaments and microtubules, by proteins called plectins.^48^ However, these filaments are not polarized and cannot support directional movement of motor proteins. The intermediate filament networks provide mechanical strength and resilience, but its contribution to mechanosensing remains poorly understood. An interaction between the intermediate filament network and actin stress fibers was recently reported in keratinocytes, which regulates their matrix rigidity sensing and downstream signal transduction.^49^ Interestingly, a correlation was established between the keratin flow and both the speed and trajectory of migrating keratinocytes, suggesting that keratins enhance persistence and migration through an interplay with actin, which is modulated by matrix adhesions.^50^ Intermediate filaments can resist tensile forces much more effectively than compressive forces and are one of the main components of the nuclear envelope. Indeed, intermediate filaments consist of polymerized nuclear lamins that contribute to the mechanical integrity of the eukaryotic nucleus.^51,52^

**FIG. 2.**
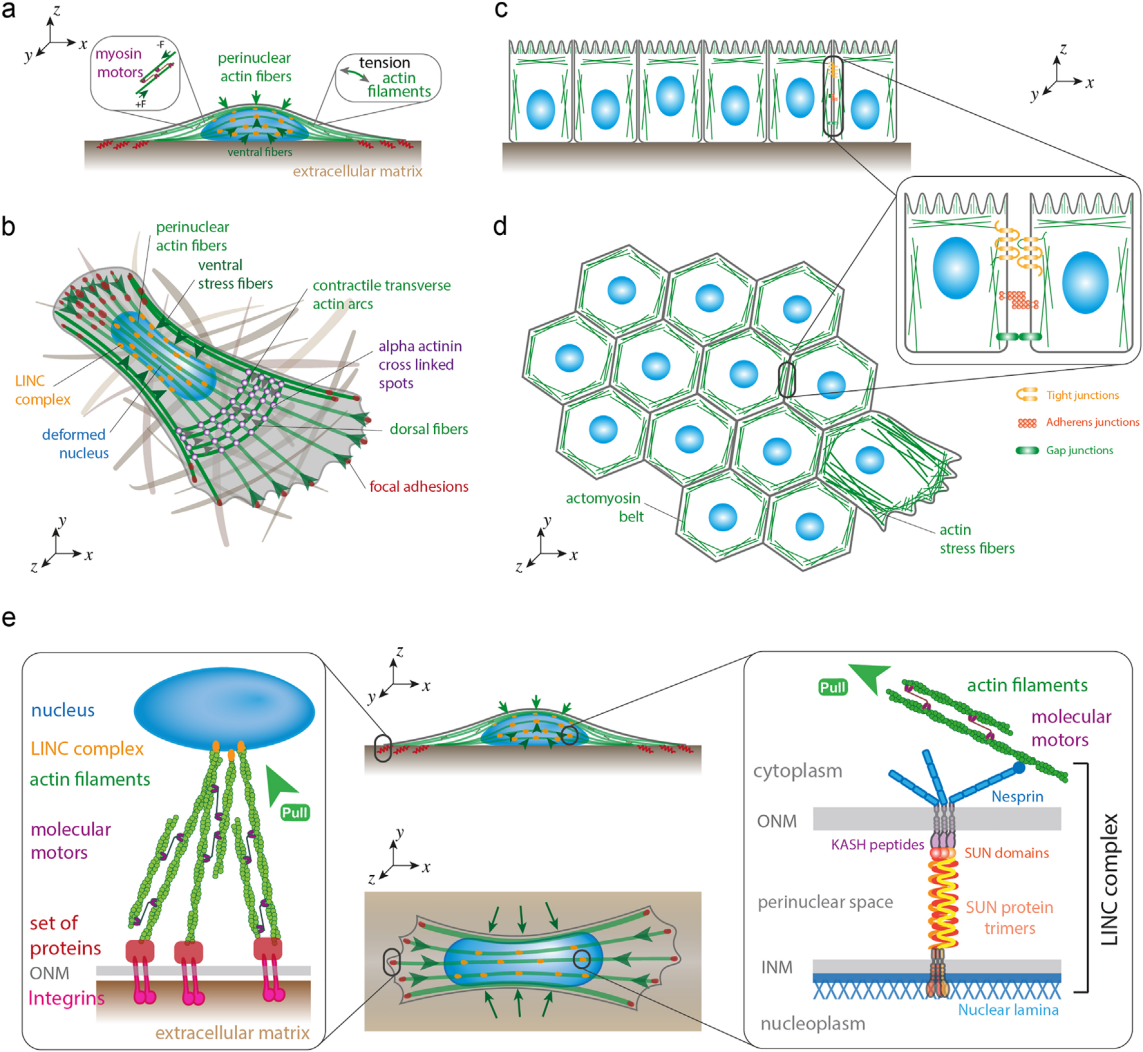
Cytoskeletal regulation of epithelial cell shape and nucleocytoskeletal coupling. (a) Actin stress fibers interact with myosin II molecular motors to form contractile actomyosin stress fibers that exert vertical (perinuclear actin fibers) or lateral (ventral actin fibers) compressive forces on the nucleus. (b) Perinuclear and ventral stress fibers interact with the nuclear lamina through LINC complexes (in orange), whereas their extremities are connected to the extracellular matrix (ECM) through focal adhesions (in red). Dorsal stress fibers do not contain myosin II and, thus, are unable to exert contractile forces, whereas transverse arcs are contractile acto-myosin bundles that are involved in the retrograde flow in migrating cells. (c) The actin cytoskeleton is organized into a belt of bundled actin filaments that runs around the apical end of the cell. (d) Tight junctions, adherens junctions, and gap junctions form an adhesion belt that encircles each of the interacting epithelial cells, while a contractile bundle of actin filaments runs along the cytoplasmic surface of the junctional plasma membrane. (e) Focal adhesions serve as a mechanotransduction hotspot to transmit forces from the ECM to the nucleus through integrins, which are transmembrane proteins connected to the cell cytoskeleton that use LINC complexes to interact with the nuclear lamina. LINC complex is a protein complex associated with both inner (INM) and outer (ONM) membranes of the nucleus that physically connect the nuclear interior with the cytoskeleton.

The dynamic regulation of the cellular shape is ensured by the spatial reorganization of the cytoskeleton and by the regulation of the cytoskeletal tension.^53^ The cooperation between actin filaments and myosin II [Fig. 2(a)], which are held together by cross-linking proteins, such as alpha-actinin or fascin, leads to the formation of stress fibers that have been grouped in different subtypes.^54^ Dorsal stress fibers do not contain myosin II and, thus, are unable to exert contractile forces,^55^ whereas transverse arcs and ventral stress fibers are contractile acto-myosin bundles^56^ [Fig. 2(b)]. Transverse arcs are curved actin bundles involved in the retrograde flow in migrating cells. Ventral stress fibers, which are considered as the main major force-generating actomyosin bundles in migrating cells, are connected to the microenvironment through focal adhesions that are the primary site of contact with the extracellular matrix (ECM). Interestingly, the formation of ventral fibers is thought to emanate from dorsal stress fibers and transverse arcs, even if the exact mechanism is still unclear.^57^ In epithelial cells, the actin cytoskeleton is organized into a belt of bundled actin filaments that runs around the apical end of the cell [Figs. 1(c) and 1(d)]. Epithelial and endothelial cells provide protective barriers to the various organs from their surroundings and help maintaining homeostasis. These protective barriers are categorized as tight junctions (TJs), adherens junctions (AJs), and desmosomes [Fig. 2(d)]. Proteins in the TJ barrier are mainly involved in the regulation of intercellular communication and paracellular transport, and based on their functions, they are classified as anchoring junctions, gap junctions, and TJ proteins. Adherens junctions form an adhesion belt that encircles each of the interacting epithelial cells, while a contractile bundle of actin filaments runs along the cytoplasmic surface of the junctional plasma membrane. The actin filaments are joined from cell to cell by transmembrane adhesion proteins called cadherins.

In many physiological processes like cytokinesis^58^ and cell migration, biochemical signals induce cell contraction through a reorganization of cytoskeletal networks. In cytokinesis, the cleavage is formed by contract forces exerted at the midpoint, while active membrane extensions are formed during cell migration such as lamellipodia and filipodia through actin polymerization [Fig. 2(d)].^59^ Recently, optogenetic experiments have shown that focal adhesion kinase (FAK; also known as PTK2) is involved in regulating the adhesion area, while the actin-bundling protein fascin is involved in regulating the cell height, suggesting that FAK and fascin work together to maintain cell shape by regulating adhesion area and height, respectively.^60^ Even if the cytoskeletal contraction is an important part of the regulation of cell shape changes, cell–substrate interactions also play a key role in mechanosensing mechanisms leading to a cellular adaptation via cell shape changes.^61^

### B. Cell shape regulates mechanotransduction pathways

Cells sense a variety of different mechanochemical stimuli and promptly react to such signals by reshaping their morphology and adapting their structural organization and tensional state. External forces applied to cells or modifications of their mechanical environment are propagated from the extracellular environment to the different organelles through cytoskeletal components [Fig. 2(e)]. Accumulating evidence supports the idea that changes of cell shape might trigger a reorganization of the three-dimensional conformation of the genome through nuclear deformation.^2^

However, identifying the signaling pathways involved in cell shape maintenance and cell mechanotransduction is a difficult task. To address this challenging question, many different techniques have been developed to control the shape of individual or small assemblies of cells in standardized culture conditions (e.g., dip pen lithography, colloidal lithography, electrospinning, etc.).^62,63^ Among these techniques, protein micropatterning is nowadays considered as the technique of choice to precisely control where and how cells adhere on culture substrates.^64^ Pioneering works using protein micropatterns to impose biochemical boundary conditions demonstrated not only that the spatial distribution of single cells is important for their survival^65^ but also that mechanical cell–matrix interactions modulate cytoskeletal tension and play a key role in the control of directional extension of the leading edge.^66^ The two-dimensional (2D) control of the cell shape with micropatterns was also very useful to better understand the mechanism governing spindle orientation, which depends on how the mitotic spindle interacts with the cell periphery.^67,68^ More recently, basic functions of human bone marrow-derived mesenchymal cells (hMSCs) were studied by using a library of topography-induced cell shapes based on 2176 randomly generated surface topographies.^69^ This work demonstrated that the relationship between cell shape and physiological response was strictly correlated, and the transcriptomics analysis revealed a tight link between cell shape and phenotype.

Cell shape changes often require modifications of cell spreading, which can result in nuclear flattening in concert with extension of the wrinkled nuclear surface. Elongation of the cell shape was shown to trigger major nuclear deformations through a spatial reorganization of ventral actomyosin fibers that exert lateral compressive forces on the nucleus^2,70^ [Fig. 2(e)]. Interestingly, nuclear elongation in response to lateral compressive forces was observed to lead to nuclear volume reduction and global chromatin condensation.^2,71^ Compressive forces exerted on fibroblast cells were observed to reduce actomyosin contractility and cause histone deacetylation, which can be correlated with chromatin condensation, reduced transcriptional activity, and activation of quiescence in fibroblasts.^72^ Using a chemomechanical model, it was recently suggested that the level of actomyosin contractility can be used as an indicator of the nucleocytoplasmic translocations of epigenetic factors for cells with different shapes and areas. Indeed, cytoskeletal forces could be transmitted through linker of nucleoskeleton and cytoskeleton (LINC) complexes [Fig. 2(e)] and then stretch the nuclear surface that can activate the opening nuclear pore complexes (NPCs).^73^ Once they are stretched, they can allow the fast transport of >40 kDa molecules, such as transcription factors—yes associated protein (YAP) or megakaryoblastic leukemia factor 1 (MLK1)—leading to further gene regulation. Interestingly, NPCs were shown to act as genome organizers and hubs for transcriptional regulation by binding both silent (i.e., heterochromatin) and active (i.e., euchromatin) domains of chromatin.^74^ Indeed, spatial constraints alter cytoskeletal organization and lamin A/C expression levels, which together lead to nuclear and chromatin dynamics.^75^ This suggests that geometric cues can directly impact nuclear functions and cell fate. For instance, it was found that MKL1-assisted actin polymerization reduced pluripotency in induced pluripotent stem cells by decreasing chromatin accessibility.^76^ MKL1 shuttling and activation are thought to be sensitive to cell shape and could be important players during geometric constraints mediated cell lineage.^77^

Altogether, these results highlight the importance of cell shape in regulating gene expression and maintaining genome integrity through active forces exerted by the actomyosin network. Interestingly, this observation at the single cell level was recently confirmed at the collective level in epithelial tissues where folding-associating gradients of cells shape and their resulting mechanical stresses direct spatially distinct biochemical responses within epithelial monolayers.^78^

### C. Actomyosin forces shape cells and tissues

Coordinated cell movements at the single cell and tissue level give rise to the right development and positioning of body precursors during embryo development.^79^ One of the most beautiful examples of collective motion is the epithelial invagination, which is a fundamental module of morphogenesis that iteratively occurs to generate the architecture of many parts of a developing organism. By changing the shape and the position of a population of cells, invagination drives processes ranging from reconfiguring the entire body axis during gastrulation, to forming the primordia of the eyes, ears, and multiple ducts and glands, during organogenesis.^80^ The execution of complex mechanisms that regulate the coordinated behavior of groups of cells has been mainly described through the identification of different signaling pathways. Accumulative evidence from mechanobiology highlights the role of forces and tension for shaping tissues during morphogenesis.^81^ Being triggered by an interplay between biochemical and mechanical signals, epithelia form complex tissues by undergoing coordinated cell shape changes, but how such spatiotemporal coordination is controlled remains an open question. One of the most challenging questions in the developmental biology is, therefore, to dissect biochemical signaling from purely mechanical cues to understand how cell shape dynamics during development is regulated and coordinated with cell fate determination.^82^

Current works consider that the mechanical aspect of this complex process is driven by two different types of parameters: the mechanical properties of cells and the external physical forces acting on them, both changing slowly over time. Based on this consideration, different morphogenetic events can contribute to epithelial morphogenesis, such as cell intercalation, cell division, cell apoptosis, and cell shape changes. One of the central mechanisms enabling cells to acquire and change their own shape is based on motor proteins that drive the generation of contractile forces within the cytoskeleton.^83,84^ During morphogenesis, the cellular cortex has been identified as an important contributor of the cell shape changes.^85,86^ The cortical zone is composed of a meshwork of filamentous actin filaments that provide a large part of the cell elastic properties.^87^ By interacting with non-muscle myosin II motor proteins, the actin network can generate contractile forces through filament sliding that serve to shape cells and tissues during morphogenesis.^88^

**FIG. 3.**
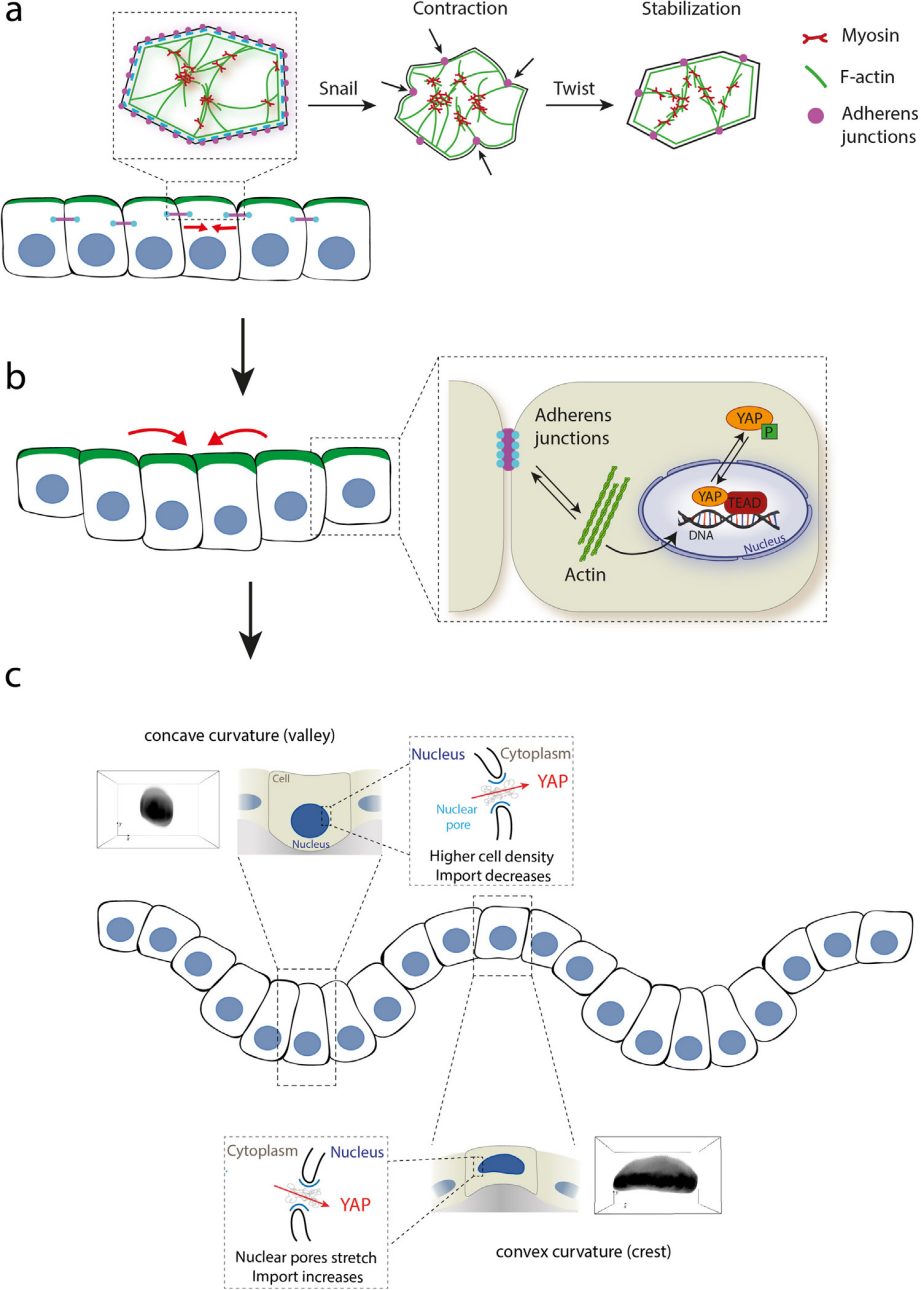
Forces driving apical constriction and epithelial folding. (a) Contraction pulses generated by the actomyosin network are initiated by Snail, whereas the contracted cell shape is stabilized by Twist. Adherens junctions assemble at the apical side into spot junctions when contraction initiates.^184^ (b) The modulation of tension at basal and apical sides drives the folding of epithelia. Tension generated at adherens junctions and actin (de)polymerization control YAP activity. The mechanical environment can influence (de)polymerization of actin filaments, which, in turn, affects tension exerted at the adherens junctions. Increases in F-actin and tension inhibit Hippo signaling and promote YAP activity.^185^ (c) Modulation of cell shape and density in response to matrix curvature changes leads to YAP nuclear export on crests and nuclear import in valleys.^67^

During morphogenesis, actomyosin contractility drives cell shape changes through apical constrictions (or cell wedging) controlled by small RhoGTPases activity,^89^ which are molecular switches that control or influence various signal transduction pathways (actin cytoskeleton, cell polarity, microtubule dynamics, or membrane transport pathways).^90^ By using multidisciplinary approaches, recent studies have shown that apical constrictions drive tissue folding in the mesoderm invagination, one of the first steps of embryogenesis.^79,89,91^ Apical contractions that drive tissue morphogenesis are not random but under the control of specific transcription factors, such as Twist and Snail, which are themselves activated by morphogens,^92^ like sonic hedgehog (SHH), transforming growth factor β (TGF-β), or retinoic acid, a metabolite of vitamin A, which is known to stimulate the growth of the posterior end of the organ.^93^ By stimulating mechanically magnetic nanoparticles loaded in the embryo, snail-dependent apex pulsations that increased apical actomyosin contractility were reproduced, demonstrating the role of apex pulsations in the apical stabilization of Myosin II [Fig. 3(a)].^94^ Cell shape changes induced by myosin contractility pulses then need to be stabilized to sustain tissue elongation. This process is mediated by formin homology domain proteins (FHOD), which interact with Rho-family GTPases and regulate the actin network organization.^95^

The global coordination of the tissue remodeling during development relies not only on the spatial distribution and anisotropy of myosin but also on the ability of forces and strains to propagate at long distances.^96^ The formation of a supracellular contractile structures of actomyosin was observed in *Drosophila* dorsal closure^97^ and avian embryo development.^98^ Interestingly, a similar supracellular actomyosin structure was observed in avian embryo gastrulation.^99^ This supracellular structure exerts and controls tissue flows that regulate the shape of the embryo. It was often reported that morphogenesis signals propagate further than the range of biochemical factors propagation.^100^ Genetically induced morphogenesis signals can, thus, be propagated mechanically by cell contractility, cell adhesion, or cell polarity. This mechanical propagation of morphogenesis signal takes place under the form of a wave of cell shape changes and Myosin II activation across the tissue.^101^ Using optogenetics to reduce cell–cell mechanical coupling, the role of tissue-scale mechanical coupling between contracting cells was demonstrated in the reduction of noise that could affect the precise localization of the morphogenetic signal.^102^

We have only begun to understand the role of cell shape change in tissue morphogenesis during development. For instance, how individual cell shape change leads to global tissue morphogenesis remains unclear. To answer this question, one must focus on the mechanisms that allow intrinsic tensile forces, which are generated by the cells themselves, to propagate within tissues. Understanding how tensile forces can shape tissues and organs during morphogenesis of the embryo or in mature epithelial and endothelial monolayers^103,104^ requires to consider the role of cadherin-based adherens junctions, where cell–cell adhesions interact with the contractile forces to generate tissue-scale tension [Fig. 3(b)].

### D. Tensile forces act at cell–cell junctions to transmit cell deformation

Epithelial tissues are highly dynamic during development and organ homeostasis due to the generation of important forces required for cell division, extrusion, or intercalation.^105–107^ The dynamic nature of epithelial tissues requires, therefore, epithelial cells to change their shape continuously and remodel their intercellular junctions. During this active remodeling, epithelial monolayers are able to remodel their cell shape and reorganize their cell–cell junctions without compromising barrier function and homeostasis.^108,109^

The ability of cells to adhere to their neighbors is a fundamental property of multicellular systems. Cell–cell adhesions are made of different junctions (adherens junctions, tight junctions, gap junctions, and desmosomes)^110^ allowing the system to maintain its cohesion, even in response to large mechanical deformations. Indeed, the remarkable tensile strength of cell–cell adhesions can, for instance, withstand a strain as much as 200% before total junction rupture.^111^ Adherens junctions are comprised of two families of transmembrane adhesive receptors: the cadherins and the nectins, which both have extracellular regions that mediate adhesion of cells to their neighbors, while their intracellular regions interact with an array of proteins that modulate their connections with the actin cytoskeleton and stimulate signaling pathways.^112^ Tight junctions are located at the border of apical and basolateral cell surface domains in polarized epithelia and act as a barrier between different cell compartments by restricting ions and solutes diffusion.^113^ Gap junctions form aggregates of intercellular channels permitting direct transfer of ions or small molecules between cells,^114^ and desmosomes are adhesive intercellular junctions connected to the intermediate filaments allowing to resist to mechanical stresses.^110^

Intercellular adhesions play a crucial role in remodeling cell shapes and force transmission between cells due to their connection to the actin cytoskeleton.^115,116^ During tissues morphogenesis, cell contact deformations are stabilized by dissipation on the minute timescale, and the turnover rate of actin filaments was found to critically affect the dissipation timescale.^116^ Indeed, most of the elastic energy is stored by deformed actin filaments, and their turnover is involved in elastic energy dissipation. Intercellular adhesion junctions are, therefore, perfectly adapted to their mechanical function. In addition to be able to resist to extrinsic tensile forces, the biology of cell–cell adhesions has evolved to sustain intrinsic tensile forces exerted by the contractile actomyosin cytoskeleton.^117^ As a result of the presence of these strong cell–cell adhesions, epithelial monolayers behave as an extensible system, and the relaxation of these adhesions leads to a contractile behavior of the monolayer. In the absence of E-cadherin, adherens junctions are perturbed, inducing an increase in the number of actin stress fibers, thus promoting a tension build-up at the basal layer.^118^ Interestingly, recent reports suggest the existence of regulatory mechanisms that can preserve the mechanical integrity of epithelial layers during the recovery of disrupted adherens junctions, suggesting that tension homeostasis of epithelial cells depends on complex mechanisms related to single cell shape and the whole tissue.^115^ Moreover, epithelial monolayers have been shown to age through the maturation of their cell–cell contacts that decrease cell expansion speed.^119^

At a larger scale, how cells sense in-plane shear forces that occur during morphogenesis and tissue growth and how these forces are spread throughout a tissue are not well understood in part due to the technical difficulty to impose in-plane shear deformation within a tissue. To close this gap, experiments were performed with a silicon device based on two parallel suspended planks: one for force actuation and the other for force sensing, which apply well-controlled shear forces to an epithelial monolayer while recording cell movements and measuring forces.^120^ By moving the actuation plank, the authors showed that the shear-induced mechanical event is relayed across the epithelium by actomyosin contraction linked through E-cadherin. Interestingly, the imbalance of forces was found to be gradually dissipated through oscillatory cell movements. In addition to spontaneously generate internal shear forces, epithelial tissues are also well known to form curved shapes adapted to their functions.^19,92,121^ However, the regulation of the three-dimensional shape of epithelial monolayers and their ability to form curved shapes adapted to their functions remain challenging to evaluate because of the lack of direct mechanical measurement. This challenge was addressed by generating suspended epithelial monolayers.^122^ Epithelial monolayers were cultured on a collagen scaffold, which was polymerized between two parallel glass plates and removed via enzymatic digestion. By unfurling these suspended epithelial monolayers via micromanipulation, the authors showed that the ability of epithelial monolayers to curl relied on the asymmetric distribution of molecular motors that generate out-of-plane stresses.

Interestingly, cumulative evidence suggests that cell–cell junctions are not only involved in physically coupling epithelial cells together and regulated the balance of internal stress within the tissue but must be also considered as an important mechanosensor involved in mechanotransduction.

### E. Cell–cell junctions mediate mechanotransduction signals

In addition to their mechanical role, cadherin-mediated cell–cell adhesions have the ability to induce a cortical spatial signal that can govern the interphase cell shape and control the mitotic spindle orientation,^123^ suggesting that intercellular adhesions are involved in mechanotransduction pathways. For instance, the local reduction of tight junction can lead to the establishment of leaks, which are associated with elongating junctions. Indeed, frequent morphogenetic movements in epithelial monolayers require elongation of cell–cell junctions to accommodate cell shape changes. It was shown recently that leaks are dynamically repaired by activations of the small GTPas RhoA, suggesting that RhoA may be activated by a membrane tension-mediated mechanosensitive pathway.^109^ It was also reported that cadherin signaling involves many pathways, including β-catenin and the YAP/transcriptional coactivator with PDZ-binding motif (TAZ) hippo signaling pathway [Figs. 3(b) and 3(c)].^124,125^ Indeed, an increase in nuclear localization of YAP was observed in the absence of E-cadherin.^118^ Recently, it was reported that stress fibers and tricellular junctions, which are two fundamental epithelial structures, can mediate the scaling between the Hippo signaling pathway and cell area, explaining how cells of different sizes collectively adapt their mechanical response to control shape and proliferation of epithelial tissues.^126^

Altogether, these examples show the key role of cell–cell junctions: the transmission of cell deformations and mechanotransduction signals. Interestingly, topological defects in epithelial tissues have been described as mechanotransduction hotspots that can govern cell death and consequent cell extrusion from epithelial monolayers.^127^ Despite the important linkage of cell extrusion to developmental, homeostatic, and pathological processes,^128^ the underlying mechanism is not well understood. An elegant way to address this problem is to model the epithelium as an active nematic liquid crystal by considering that apoptotic cell extrusion is provoked by singularities in cell alignment in the form of comet-shaped topological defects.^129^ In Sec. I F, we will summarize recent findings about the understanding of mammalian biological tissue as active nematic materials and how biological functions arise from these properties. For better understanding of nematic behaviors of cytoskeletal systems at the molecular level, interested readers can refer to excellent reviews in the field.^130–132^

### F. Exploiting physics of liquid crystals to connect cell shape changes to orientational order in cell aggregates

Force transmission, through cell–cell contacts, provides a directional guidance for the transfer of biochemical signals, such as extracellular signal-regulated kinase (Erk) waves, into specific location.^133–135^ This process can give rise to collective motion and the emergence of order that is established and maintained across a range of scales.^136,137^ Interestingly, this order is based on the anisotropic shape of the cells that adopt an elongated morphology, resulting in a well-ordered orientation field within the tissue.^118,127,138,139^ The anisotropic cell shape prompts cells to spontaneously align in ordered domains to lower their collective energy, leading to an analogy with liquid crystals, which defines an intermediate phase between solid and liquid that flows like a liquid but maintains some of the ordered structure of crystals, in particular, with nematics liquid crystals which exhibit aligned rod-like particles that present head-tail symmetry in their orientation.^132^ The physical laws governing phase transitions and dynamic of liquid crystals have been reported in various subcellular processes and were related to an interplay between cell shape and the spatial organization of the cytoskeleton, especially for rigidity-sensing mechanisms.^140,141^ Indeed, the transition between circular and stationary phenotype on soft substrates to an elongated and motile phenotype coincides with the isotropic-to-nematic transition of the actin cytoskeleton that forms contractile stress fibers above a transition stiffness resulting in polarized and elongated cells.^141–143^ Based on active nematic theory, it was reported very recently that the self-organization of myoblasts around integer topological defects, namely, spirals and asters, triggers localized differentiation and, when differentiation is inhibited, drives the growth of cylindrical multicellular protrusions.^144^

Interestingly, the physics of liquid crystals can also serve to design new biomaterials for controlling cellular organization. It has been shown recently that prepatterned micrometer-sized ridges can be exploited to control alignment of fibroblast and epithelial cell layers to form stable full-integer topological defects.^145^ Remarkably, it was shown that such micrometer-sized topographical patterns that are imposing defects of integer topological charge can control the activation/deactivation of mechanotransduction by triggering nuclear/cytoplasmic YAP translocation at topological defects with positive and negative charges and in different cell types. This work demonstrates the relevance of developing new bioengineering tools combined with mechanical assays to provide further insights into the morphobiological mechanisms that regulate the pathophysiological behavior of epithelial cells.

### G. Inspired bio-engineering tools: mechanobiology-on-a-chip

**FIG. 4.**
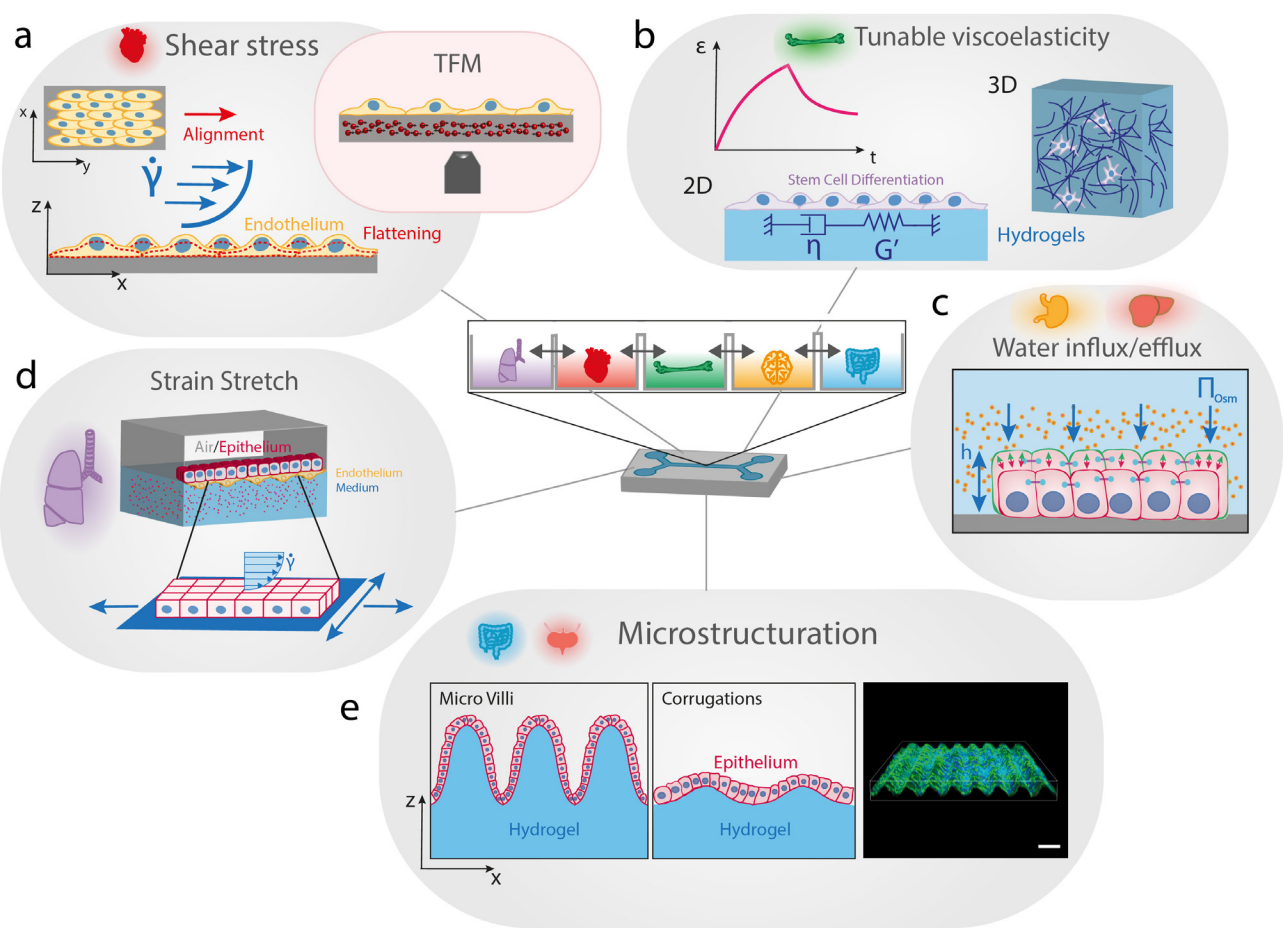
Advanced bioengineered platforms integrating mechanobiology assays. (a) The shear stress exerted on endothelial cell monolayers can trigger multiple cell shape changes, such as elongation and flattening. Traction force microscopy (TFM) can be integrated to microfluidic devices by incorporating micrometric beads into an elastic elastomer to provide the cellular traction force field in response to a given shear stress. (b) Time-dependent mechanical properties of native tissues are a crucial determinant of cell shape and fate. The next generation of synthetic matrices must have well-defined stiffness and tunable viscoelastic properties, with variable relaxation times. 2D and 3D encapsulating hydrogels are useful synthetic matrices for studying the influence of viscoelasticity on epithelial cell fate. (c) Aqueous solutions of polymers can be used to modulate the osmotic pressure and trigger cell shape changes via volume expansion/diminution. (d) Microfluidic devices can integrate air/cell and liquid/cell interfaces and reproduce complex strain fields by incorporating uni- or multi-axial stretchers. Stretchable microfluidic devices with dual interfaces allow to mimic the microenvironment and the mechanical loading of epithelial lung tissues. (e) Villi in the small intestine and corrugations of many epithelia impose curvature changes at the cellular scale. Soft hydrogels can be shaped to form corrugations that reproduce variations in local curvature. Sinusoidal culture surfaces allow to study how epithelial monolayers adapt and sense cell-scale curved topography.

*In vivo*, cells and tissues are exposed to diverse mechanical stimuli that can modulate their functions though cell shape changes. Microtechnology has started to provide new approaches for reproducing *in vitro* the physico-chemical complexity of native tissues, thus for studying mechanotransduction pathways in more realistic conditions. More recently, advanced culture platforms have emerged and combine complex physico-chemical environments with dynamic mechanical loadings to recapitulate key mechanobiological aspects of epithelial tissues in the different organs (Fig. 4). A wide range of dynamic mechanical cues can be modulated into these microfluidic platforms for mechanobiology (shear stress, stretching, stiffness, pressure, etc.) to correlate cellular deformations to (dys)functions.^146^

#### 1. Shear stress

Microfluidics is a high throughput technique, which requires very small reaction volumes, and can be designed to closely reproduce flow conditions observed *in vivo* [Fig. 4(a)]. In addition to reproduce various *in vivo* flow conditions, microfluidics is widely used to improve single-cell workflows, from cell sorting to lysis, to sample processing and readout.^147^ Thanks to a fluid perfusion inside micrometer-sized channel, microfluidics allows to apply well-controlled shear stresses on cell cultures, which plays an important role in endothelial cell behavior.^148^ Fluid shear stress causes not only blood vascular endothelial cells to align in the direction of flow^149^ but also vascular smooth muscle cells to align perpendicular to the direction of flow.^150^ In the local mechanical environment of angiogenesis, endothelial cells are subjected to various external stimuli, including shear stress changes and matrix stiffness modifications. These mechanical stimuli influence the behavior of endothelial cells, modulating their ability to generate traction forces. In addition, shear stress has been shown to modulate osteoblast cell and nucleus morphology. This shear stress induces actin reorganization around the nucleus, which reduces both cell and nucleus volume.^151^ The next generation of microfluidic devices will be capable to recreate mechanobiological relevant cellular microenvironments.^152^ For instance, new insights about mechanotransduction pathways on endothelial cells were recently reported by combining microfluidic systems with traction force microscopy (TFM).^153^ Cellular traction forces were suggested to act as possible effectors activated by mechanosensing to mediate matrix remodeling, demonstrating that the use of TFM to study mechanotransduction in angiogenesis is highly relevant.

#### 2. Viscoelastic properties of the matrix

Since the past few decades, the role of the stiffness of the cell microenvironment has been studied extensively.^142,154,155^ In their seminal work, Engler *et al.* showed that the differentiation of human mesenchymal stem cells (hMSCs) can be directed by modulating the elastic modulus of 2D polyacrylamide hydrogels substrates.^156^ However, the native ECM is not linearly elastic and exhibits a time-dependent response to loading, as many other viscoelastic materials. Matrix viscoelasticity regulates fundamental cell processes and can even promotes specific behaviors that cannot be observed with traditional elastic materials.^157^ Charrier *et al.* used viscoelastic polyacrylamide hydrogels to reproduce *in vitro* time-independent and time-dependent mechanical properties of healthy and pathological tissues.^158^ The authors showed that viscous dissipation in biological tissues can be considered as a determinant of cell phenotype and tissue homeostasis. Despite these efforts, the physiological relevance of most experimental approaches using 2D culture substrates was limited. Recently, the development of novel culture substrates with a controllable rigidity has enabled the extension of these studies into three-dimensional (3D) environments that more accurately mimic the biomechanical and chemical cues that cells experience *in vivo* [Fig. 4(b)]. For instance, a 3D gelatin methacryloyl (GelMA) hydrogel with a continuous stiffness gradient (from 5 to 38 kPa) was introduced to recapitulate physiological stiffness conditions.^159^ By encapsulating human adipose-derived stem cells (ASCs) into GelMA hydrogels of various rigidities, they investigated the ability of ASCs to deform the surrounding matrix through the cell volume adaptation. Low-stiffness regions (∼8 kPa) increased cellular and nuclear volumes and enhanced mechanosensitive protein localization in the nucleus, whereas high stiffness regions (∼30 kPa) decreased cellular and nuclear volumes and reduced mechanosensitive protein nuclear localization. Cell volume adaptation as a function of 3D matrix stiffness could be, therefore, a good candidate to control stem cell mechanotransduction and differentiation. The impact of hydrogel stiffness on differentiation of human adipose-derived stem cells was recently studied using microspheroids. It was demonstrated that the stiffness of gelatin-based hydrogels (from ∼0.5 to 7.3 kPa) affects proliferation and differentiation of microspheroids formed from telomerase-immortalized human adipose-derived stem cells (hASC/hTERT).^160^ Very recently, alginate hydrogels were used to show that viscoelasticity and protein ligand density promote the viability and proliferation of human induced pluripotent stem cells (hiPSC). Furthermore, they reported that actomyosin contractility regulates lumen formation, which is accompanied by nuclear export of YAP. Altogether, these works showed that encapsulation of stem cell microspheroids in gelatin-based hydrogels has a promising potential for bioengineering applications and to build advanced organoids.

#### 3. Water influx/efflux

In addition to its complex rheological properties, cell surrounding is an aqueous environment, which is yet often considered as a passive arena. Recent studies have accumulated evidence to show that considering proteins as the leading players of biological functions is not the complete picture.^161^ Indeed, recent studies and decades of previous works suggest that active fluxes of water and small molecules can play essential roles during cell shape changes and in the generation of forces. For instance, ion flux was shown *in vivo* to mediate cell volume changes that contribute to asymmetric cell shape changes in Kupffer's vesicle, a transient organ with a fluid-filled lumen.^162^ By using polyethylene glycol (PEG) to apply well-controlled osmotic pressure conditions [Fig. 4(c)], Guo *et al.* showed that cell volume decreases through water efflux, causing subsequent changes such as the alteration of mesenchymal stem cell differentiation.^163^ A similar strategy was used to demonstrate that cadherin-based junctions must be considered as a key player of the response to osmotic stress changes in epithelial cell clusters, which is of particular interest for a better understanding of metastatic breast cancer progression.^164^ Very recently, a mechano-osmotic model was introduced to describe how the cellular volume is regulated within 3D clusters.^165^ Interestingly, gap junctions were found to play a crucial role in amplifying spatial variations in cell volume, which may provide new insight in breast cancer progression. These studies highlight the importance of considering cellular volume changes and demonstrate that mechanobiology-on-a-chip and multicellular spheroids can be considered as complementary and promising experimental models to bridge the gap between *in vitro* and *in vivo* conditions.

#### 4. Substrate strain

*In vivo*, cells are exposed to stretching forces exerted by deformation of the matrix or by neighboring cells through cell–cell adhesive interactions. Many cellular processes of lung epithelial cells, contractile cardiac, and muscle cells are regulated by cyclic stretch and relaxation steps.^166–169^ In addition, stretching of brain cells is a hallmark of traumatic brain injury (TBI).^170^ During last few years, different strategies have been employed to engineer stretching devices that apply different axial strains to cell cultures. Indeed, many *in vivo* situations require to reproduce *in vitro* complex strain fields because mechanical stresses in organs or tissues (e.g., lung alveoli, bladder, or heart) are often complex and multiaxial.^167,171^ Most of the previous works have developed uni- or multiaxial stretchers to mimic physiological or pathological conditions [Fig. 4(d)]. Stretching devices allowed to understand the role of transmembrane integrins in diffuse axonal injury^172^ and how mechanical injuries of neurons induce tau mislocalization to dendritic spines and tau-dependent synaptic dysfunction.^173^ More recently, Shimizu *et al.* have developed an *in vitro* 3D vascular system that combines fluidic shear stress and stretching stress, which are both necessary to mimic the complex mechanical environment of blood vessels.^169^ By culturing endothelial cells under perfusion and simultaneously applying stretch deformations, they demonstrated that stretchable microfluidic systems are useful tools for understanding the mechanotransduction pathways in vascular tissues. Sophisticated stretch/strain devices based on microfluidic chips could be very helpful for studying the role of cadherin-based adhesive interactions, and robust co-culture models would also certainly benefit from further research.^174^

#### 5. Toward the third dimension

The attempt to grow cells in 3D showed great promise as the third dimension could allow to better recapitulate the physiological environments of most human tissues.^175^ However, only a few laboratories had setup robust 3D techniques despite many limitations of traditional 2D culture systems to mimic the complex cell–cell and cell–matrix interactions that occur in native tissues.^176^ Most biological surfaces are not flat and possess invaginations, folds, or wavy morphologies. The geometric form and biological function of 3D tissues are inherently linked together at all scales. For instance, crypts and villi of the small intestine provide a large surface area for exchange, improving the absorbance function [Fig. 4(e)].^11^ To date, only a few studies have investigated how cells react to mechanical stresses emerging in reaction to this external curvature constraint. Therefore, the relationship between curvature and biological function in epithelial tissues remains largely unexplored.^15,177^ To answer this question, some groups have started to engineer artificial culture platform that replicates 3D out-of-plane curvatures and can realistically mimic complex *in vivo* structures, such as the villification of the intestine.^178,179^ Using sinusoidal culture surfaces to probe specifically how individual cells react to cell-scale curved topography, it was shown that cells avoid convex regions during their migration, position themselves in concave valleys, and that substrate curvature differentially regulates stem cell migration and differentiation.^180^ In both cases, it was suggested that nuclear deformations play an active role in the cellular response to local curvature changes. Recently, the role of the substrate curvature was addressed at the tissue level by studying epithelial cell monolayers on engineered soft wavy hydrogels.^181^ Substrate curvature was found to affect monolayer thickness, which is larger in the valleys than it is on crests, demonstrating that cells may sense curvature by modifying the thickness of the tissue. In addition, curvature was observed to govern the spatial distribution of yes associated proteins (YAP) via nuclear shape and density changes [Fig. 3(c)] and to induce significant variations of lamins, chromatin condensation, and cell proliferation rate in folded epithelial tissues. Original approaches took inspiration from the bending motion of the tongue to engineer a bio-inspired platform where the bending properties of thin elastic membranes covered with myoblasts serve as a strength assessment to show that Duchenne muscular dystrophy (DMD) myoblasts fail to self-organize their actin cytoskeleton in response to ECM cues.^182^ An elegant self-rolling technique was recently introduced to study how epithelial monolayers adapt to a rapid and anisotropic change of curvature, showing that the primary cellular response is an active and transient osmotic swelling.^183^

## II. FUTURE PERSPECTIVES

We have only begun to understand the role of cell shape change in epithelial tissue homeostasis and morphogenesis. Recent achievements and advancements of mechanobiology indicate that the shape of epithelial cells can be understood as the result of the interplay between actomyosin contractility and intercellular adhesions, and that both do not act independently but are functionally integrated to operate on molecular, cellular, and tissue scales. The ability to sense mechanical forces at the cellular level demonstrates that the cellular response is governed by the distribution of mechanical forces throughout epithelial tissues. Local variations in cell shape owing to variations of tensile forces acting at cadherin-based adherens junctions, patterns of cell traction forces, or mechanical changes of the cell microenvironment can influence embryogenesis and tissue pattern formation in epithelial systems. The remodeling of cytoskeletal components can not only alter the local cell shape but also generate contractile forces that drive the motion and extension of collective populations. Interestingly, emerging evidence suggests that additional parameters must be considered such as cell–matrix interaction, cortical tension, and cellular viscoelastic properties.

Even if several signaling pathways and effector molecules have been identified, it is clear that feedback loops exist between cellular mechanics, protein expression, and genomic activity. To further identify the molecular mechanisms of these feedback loops, future studies that aim to understand the specific structures and molecules that mediate the cascade of multiscale events during mechanotransduction must consider the role of internal and external forces and the multiscale architecture of epithelial tissues. Knowing that the nuclear shape and function are intimately regulated by the cell shape change, we envision, therefore, that the field of mechanobiology will help to answer ground-breaking questions such as how individual cell shape changes can lead to global tissue morphogenesis. This question can be addressed by analyzing eventual correlation between tissue morphogenesis and cell shape changes or by using optogenetic approaches to modulate in space and time individual cell shape. Other open questions are, for instance, how nuclear deformations trigger intracellular events that promote cell behavior and how physical stress waves propagate during epithelial expansion? Success in answering these open questions relies on the complex interplay between mechanics, chemistry, and biology at all size scales and on the development of innovative *in vivo* and *in vitro* mechanobiology assays. In addition, the combination of theoretical modeling with quantitative measurements obtained will be a powerful approach to understand the regulation mechanisms of cell shape during epithelial events such as tissue morphogenesis, folding, self-healing, intercalation, or extrusion. Collaborative interaction alliances with bioengineers, experimental and theoretical physicists, and molecular biologists are the key to a successful development of groundbreaking methodologies for epithelial cell mechanobiology.

## III. GLOSSARY

Acto-myosin contractility: Contractile activity of the actin cytoskeleton mediated by non-muscle myosin II-A and II-B motor proteins. Actomyosin contractility is responsible for traction forces exerted on the substrate at cell–matrix adhesions.

Buckling: Collapse of a structural component under compression.

Chromatin condensation: Dramatic reorganization of the long thin chromatin strands into compact short chromosomes that occurs in mitosis and meiosis.

Compression: Force met by the resistance of a material against a decrease in its length.

Extrinsic forces: Outward mechanical forces transduced to the cell.

Extrusion: Process preventing the accumulation of unnecessary or pathological cells. Cell extrusion can be triggered by apoptotic signaling, oncogenic transformation, and overcrowding of cells.

Fluid shear: Drag force oriented parallel to the surface and caused by the flow of fluid past a material.

Gyrification: Mechanical process forming the characteristic folds of the cerebral cortex.

Hydrogel: Three-dimensional network of crosslinked hydrophilic polymers that can swell in water and hold a large amount of water while maintaining their structure due to the presence of chemical or physical cross-linking of individual polymer chains.

Intrinsic forces: Contractile forces produced from inside the cell and mediated by the actomyosin cytoskeleton.

Mechanical homeostasis: Emerging mechanobiology concept related to the natural tendency of tissues to maintain a preferred mechanical state under environmental perturbations by modulating the delicate balance among growth, remodeling, and degradation.

Mechanobiology: Research field at the interface of biology, physics, and bioengineering that focuses on the study of physical processes by which mechanical signals can shape cell and tissue behaviors.

Mechanotransduction: Mechanism by which cells convert external mechanical stimuli into a biochemical output.

Mechanosensing: Process of a cell sensing mechanical signals provided by its environment.

Molecular motor: Class of proteins capable of converting energy into work. Myosin is a common molecular motor, which hydrolyzes adenosine triphosphate (ATP) into adenosine diphosphate (ADP) and exerts a mechanical work on an actin filament using chemical energy.

Optogenetics: Experimental method combining optics and genetics to control in space and time well-defined molecular events in cells or tissues.

Organs-on-a-chip: Microfabricated devices designed to recapitulate the function and morphology of an organ.

Persistence length: Basic mechanical property quantifying the bending stiffness of a polymer. It is the distance (expressed in meters) along a rigid polymer over which the direction changes statistically.

Prestress: Existing internal stress usually generated by myosin-II mediated actomyosin contractility.

Protrusive force: The force developed at the leading edge of the cell for lamellipodial movement and generated by the growth of actin filaments pushing against the cell membrane.

Retrograde flow: The movement of actin filaments rearward with respect to the substrate toward the center of the cell. Retrograde flow is generally in a direction opposite to the movement of the cell.

Stiffness: Ability to resist deformation in response to an applied stress. Stiffness or elastic modulus has the same unit as stress (N/m^2^ or Pa).

Strain: Deformation per original length due to the applied stress. Strain is dimensionless.

Stress: Force applied per unit area. The unit is Newton per square meter (N/m^2^) or Pascal (Pa).

Tension: Force met by the resistance of a material against an increase in its length.

Tissue morphogenesis: Process of organizing the spatial distribution of cells during embryonic development.

Traction forces: Forces exerted by a cell at the cell/matrix interface determined from the displacement field at the surface of the matrix. It is the force the cell generates against the substrate in order to move forward. Tractions forces have usually the unit of a stress (N/m^2^ or Pascal).

Viscoelasticity: Rheological property of complex materials that exhibit mechanical properties intermediate between elastic solids and viscous liquids when undergoing deformation.

Viscous dissipation: Irreversible transfer of mechanical energy to heat by the flow of a viscous fluid.

## Data Availability

Data sharing is not applicable to this article as no new data were created or analyzed in this study.
